# Mr. Bucknill on Poisoning by Arsenic

**Published:** 1813-01

**Authors:** William Bucknill

**Affiliations:** Nuneaton


*To the Editors of the Medical and Physical Journal.*

GENTLEMEN,

ON reading your last Number, I perceive some cases given by Mr. Davis, where arsenic had been taken; which reminds me of a person whom I was called to nearly three years since, who had swallowed an ounce of that pernicious mineral: which case, if you think it worth recording, you will please to give a place in your Journal; not that I expect much practical information can be derived from its publication, but it will show that a large quantity of arsenic may be taken into the stomach without producing fatal effects.

A woman, at her usual time of going to bed, took an ounce of arsenic in a cup of tea, which she supposed to be crem tart. In a few minutes after, she felt a burning sensation in her fauces and stomach, which very shortly was succeeded by violent vomiting, continuing the whole of the night; had an insatiable desire for drinking, which induced her to take large and repeated draughts of warm water. About four hours after taking the mixture, she was attacked with a most excruciating pain in her bowels, soon followed by a diarrhœa, and the acrimony of the discharge excoriated the anus. About eight o'clock in the morning, the mistake being discovered, I was sent for, and found the woman laboring under the above symptoms, with a pulse scarcely perceptible, and in a cold clammy sweat. I ordered her to take as much broth as she could get down, and have oily glysters given frequently; towards evening the inflammatory action came on; the case was then treated as gastritis. In two or three weeks the woman recovered, except in having occasional attacks of cramp in the stomach, which returned for upwards of twelve months, and then gradually left her. At this time she is in perfect health.

I also observed, in your last Number, a Practical Physician attempting to ridicule Dr. Wilmer's treatment in a supposed case of catalepsy. Allow me to make a few remarks, particularly as one of the medical men named is now no more, and the other (Dr. W.) is at Bath, for the purpose of recruiting his health. It is more from the mode of attack, than from any other motive, that I have been induced to mention the subject. Criticism, when candid and just, is generally useful, as it tends to the elucidation of facts, but I never can give any one credit for a desire to improve science who shields himself under an anonymous title. The case in question I knew nothing of till I saw it in your Journal. It is my opinion, that many of the diseases incident to children may be referred to a derangement either of the hepatic system or the alimentary canal; and that the convulsions, in the case alluded to, were entirely symptomatic. But P. P. cavils at the fear excited by the perseverance in the use of calomel, and begs to ask Dr. W. whether he ever saw, or ever heard of, any child whose mouth was effected by calomel, at that age, or even years after? I do not know what P. P. may have seen in his practice, but I had once a case of a child under five years old where calomel was given, conjoined with other purgatives, which, not going off by stool, brought on a complete *ptyalism;* and I can safely assert, that the salival discharge exceeded a pint in 24 hours. Having but little time, and less inclination, for controversy, I do not wish to resume the subject; neither shall I, unless P. P. gives his real name: and, as he never heard of a child being salivated, I trust the novelty of the case will give him ample satisfaction for my remarks. I am, Gentlemen,

Your's, most obediently,

*Nuneaton, Nov.* 12, 1812. WILLIAM BUCKNILL.

